# Supplementary figures and images for: Fast and flexible linear mixed models for genome-wide genetics
Source: PLoS Genet. 2019 Feb 8;15(2):e1007978. doi: 10.1371/journal.pgen.1007978 (PMC6383949; doi:10.1371/journal.pgen.1007978)

**a**

seconds per iteration

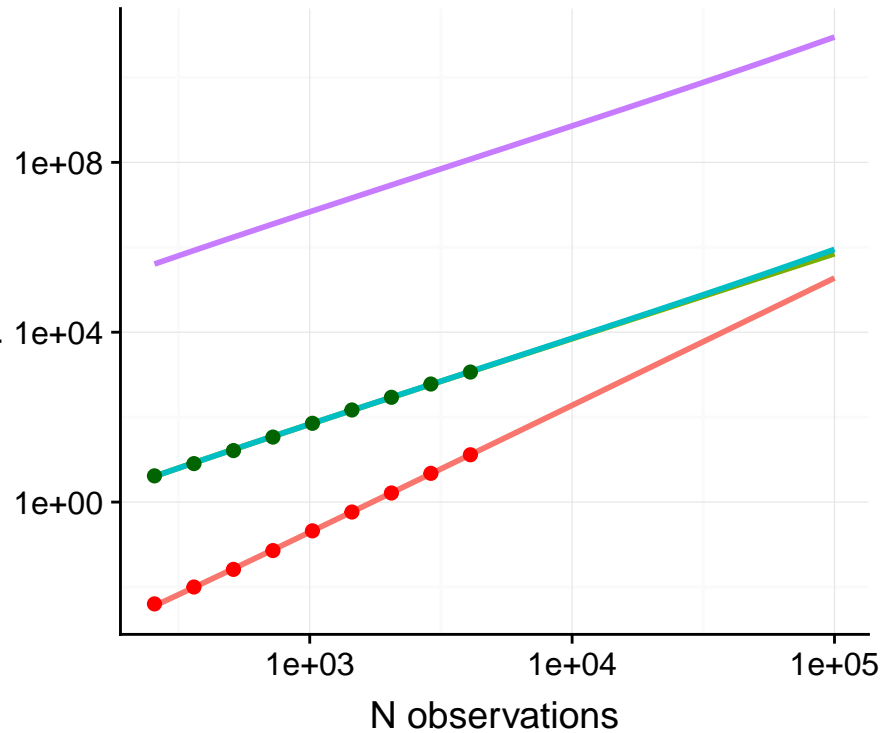**b**

MB for Cholesky matrix

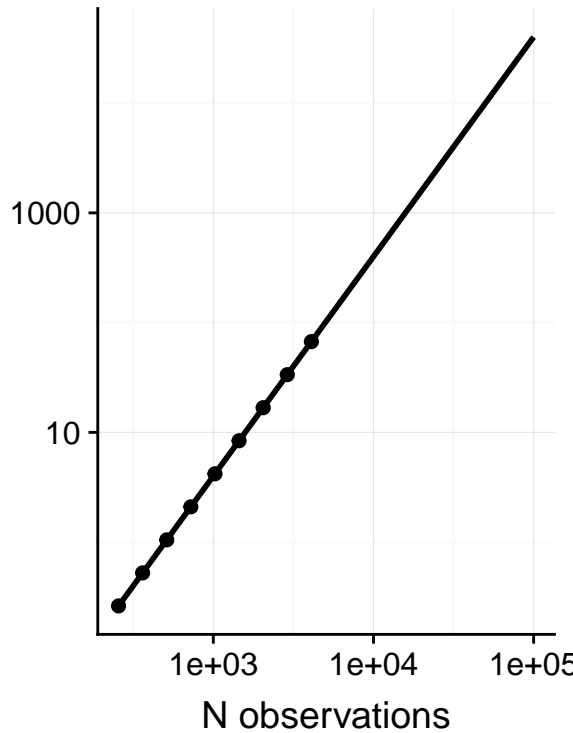

Supplement: S1 Fig — (a) Computational times for the most costly steps of typical mixed model fitting algorithms: inverting an n × n covariance matrix (generally using a Cholesky decomposition), and multiplying the inverse matrix by an n-vector (i.e. a vector of marker genotypes). The red curve shows the time required for a Cholesky decomposition using the base R function chol as a function of n. The green curve shows the time required for a Cholesky matrix-by-marker matrix multiplication with 1 × 10^5^ markers, as a function of n. The blue curve is the sum of the Cholesky decomposition and matrix-vector multiplication operations for a single grid-cell in Grid-LMM with 1 × 10^5^ markers. The green and blue curves are barely distinguishable across most of the range because the Cholesky decomposition is generally not limiting. The purple curve would be the expected time for a separate Cholesky decomposition and matrix-vector multiplication for each marker in a GWAS with 1 × 10^5^ markers (i.e. the cost of a typical exact-LMM method such as LDAK for a single iteration). Both the Grid-LMM and LDAK times are per-iteration. Grid-LMM requires this time at each grid cell. LDAK requires multiple iterations for the REML optimization separately for each marker. Generally, Grid-LMM will evaluate more grid cells than LDAK requires iterations per test. However this will not cause a reversal in the relative time requirements unless a very large grid is used. (b) Memory requirements for storing an n × n Cholesky matrix as a function of sample size. In both panels, the curves were extrapolated based on tests with n between 256 and 4096 (actual times shown with points). All timings were estimated using base R functions. (PDF) [file pgen.1007978.s001.pdf]

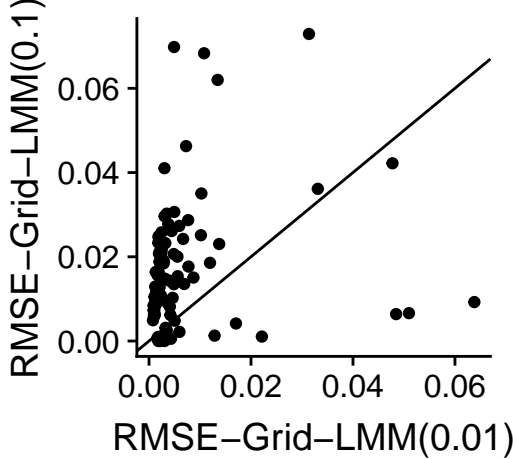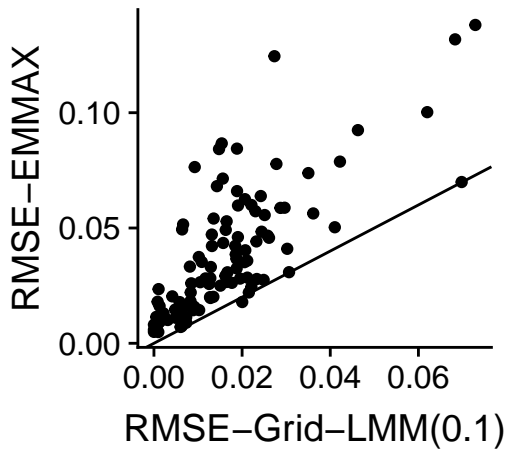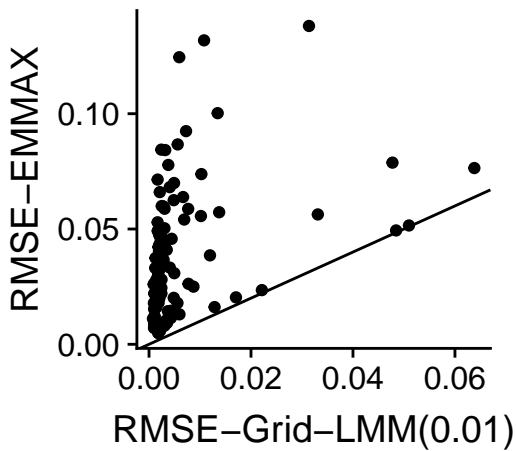

Supplement: S2 Fig — GWASs were run for each phenotype using 216,130 markers and up to 199 accessions, with a single random effect controlling for additive genetic relationships among lines. For each phenotype (represented as a single point in the plots), we compared the exact Wald-test -log10(p) calculated by GEMMA to p-values calculated by the approximate methods EMMAX, and Grid-LMM using either the naive approach with a complete grid of size 0.1 h2-units, or the fast heuristic algorithm Grid-LMM-fast with a fine grid size of 0.01-h2 units. Grid-LMM p-values were always at least as accurate (as measured by root mean-squared-error, RMSE) as those calculated by EMMAX. Specifically, p-values calculated with a fine grid-size of 0.01 (using the fast algorithm) were nearly indistinguishable from those of GEMMA, except in the rare cases where the REML surface was not unimodal. This was generally restricted to a small subset of rare markers with small-moderate effect sizes, and only occurred for a few traits. In these cases, the complete—but more coarse—grid search of Grid-LMM with step sizes of 0.1 was more accurate. (PDF) [file pgen.1007978.s002.pdf]

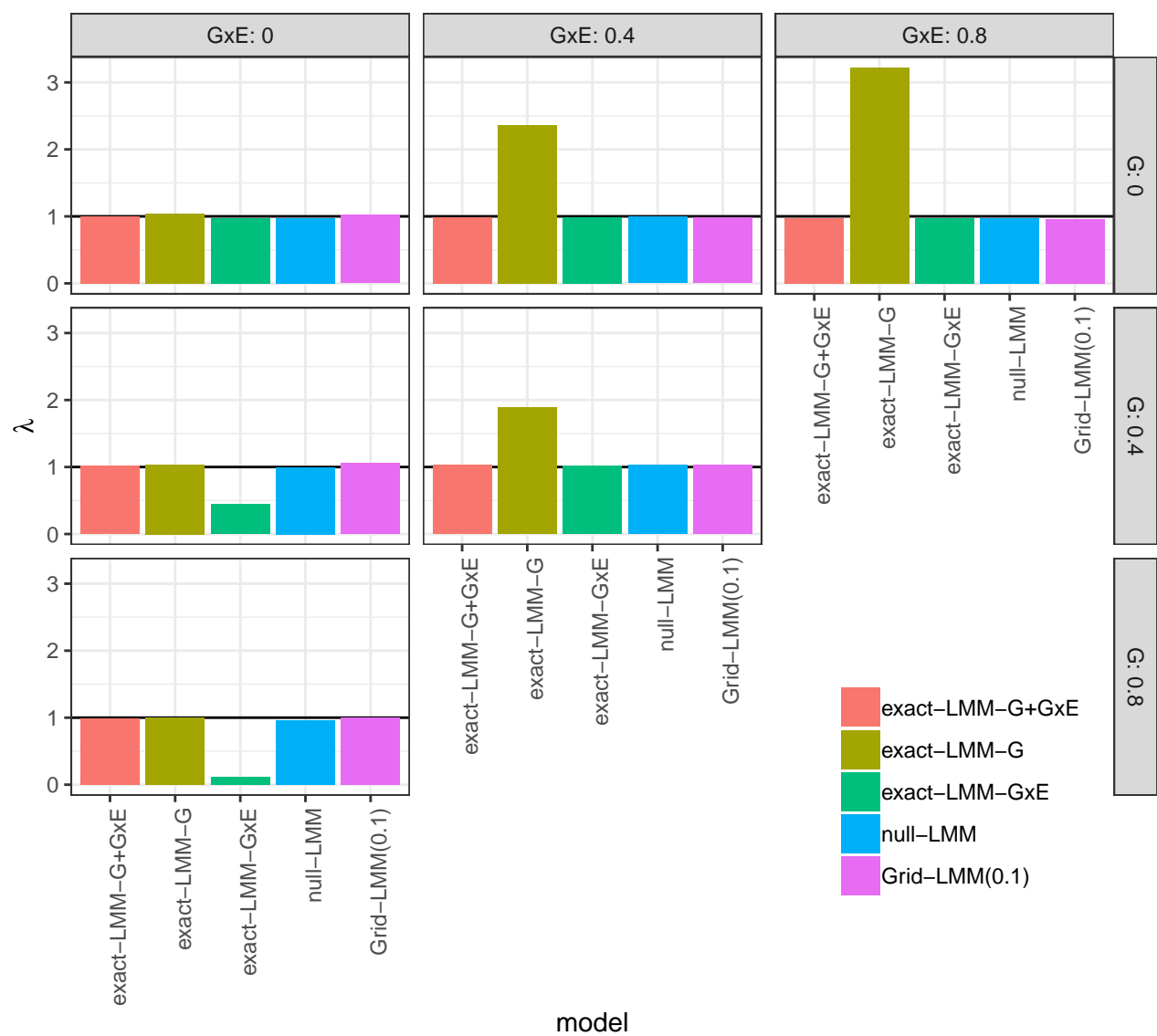

Supplement: S3 Fig — Simulated datasets were created based on the Atwell genotype data and the G×E analysis. We randomly selected 10,000 markers, generated simulated data with different proportions of additive (G) and gene-environment interaction (G× E) variation for each marker, and calculated Wald F-statistics for an interaction between the marker and the environment. Bars show an estimate of genomic control inflation factors [1] for each of the following five methods. exact-LMM-G+GxE is an exact LMM algorithm fit with LDAK. This model included both random effects and the marker effect. At a genome-wide scale, it is very slow, with computational complexity O(ptn3). exact-LMM-G and exact-LMM-GxE are exact LMM algorithms, similar to GEMMA, which included only one random effect and the marker effect. null-LMM is an approximate method similar to pylmm that conditions on variance components estimated under a null model with no marker effect. It was run with both random effects. Grid-LMM was run with a grid size of 0.1 h2-units and included both random effects and the marker effect. The λ values were calculated as the ratio between the median value of the the F-statistics returned by each model and the median value of a F1,316−4 distribution. The horizontal line shows the expected value λ = 1 under the true model. (PDF) [file pgen.1007978.s003.pdf]

Power with  $\alpha = 2e-7$

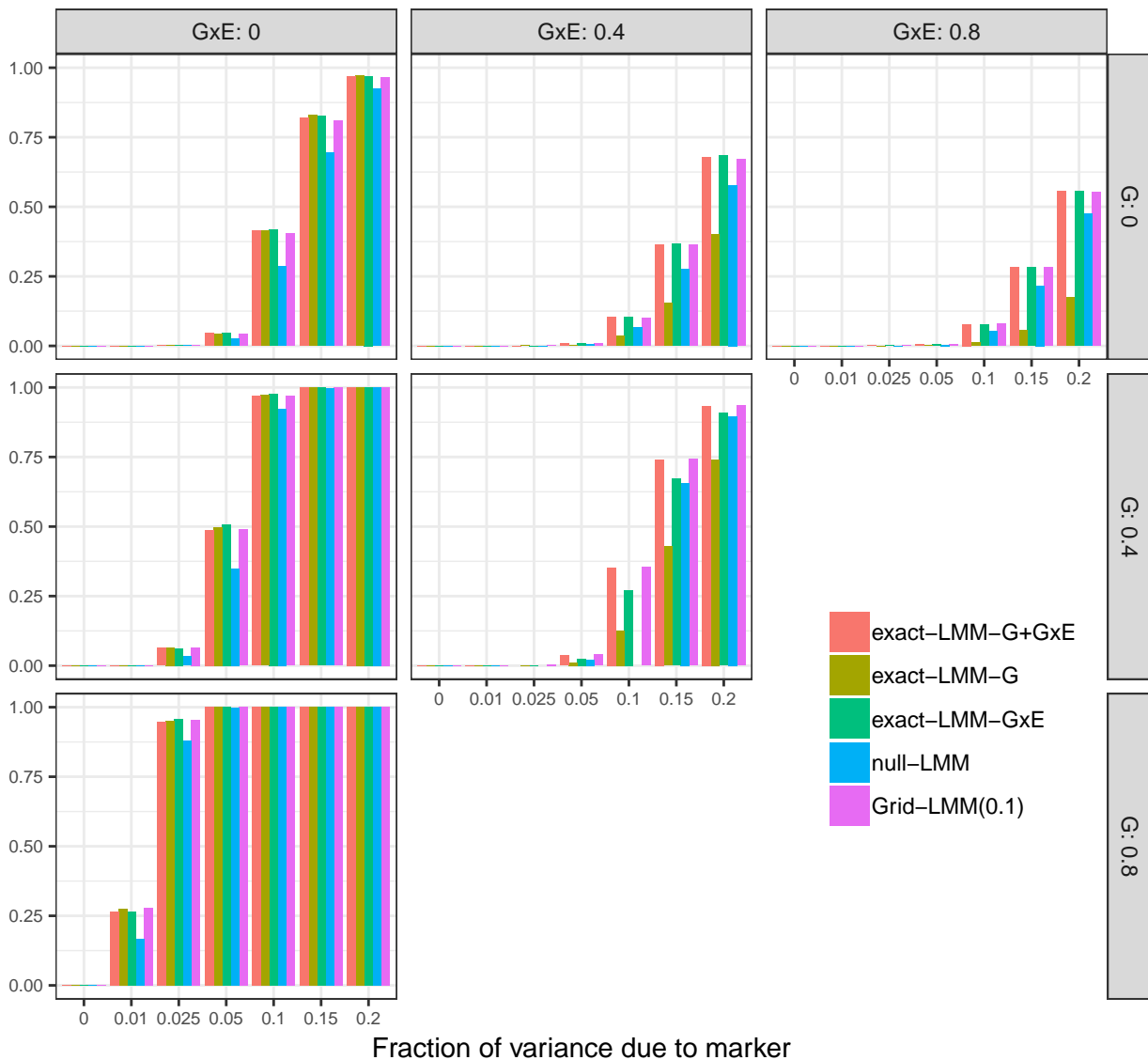

Supplement: S4 Fig — Bars show the genome-wide power for randomly selected SNPs in the Atwell genotype data under simulations with different proportions of additive (G) and gene-environment interaction (G×E) variation with different marker effect sizes. Simulations were generated as described in S3 Fig, and included only a single marker with zero main effect and G×E effects scaled to a defined percentage of the phenotypic variation. The remaining phenotypic variation was simulated from a multivariate normal distribution constructed by appropriately weighting the additive relationship matrix, the G×E covariance matrix, and the uncorrelated residual variation. Each simulation was run separately for 10,000 randomly selected markers. Wald F-statistics from each method were normalized by dividing by the genomic control inflation factor computed for Figure, and then p-values were calculated and compared to the Bonferroni corrected threshold P = 2 × 10^−7^ to determine significance. (PDF) [file pgen.1007978.s004.pdf]

**a**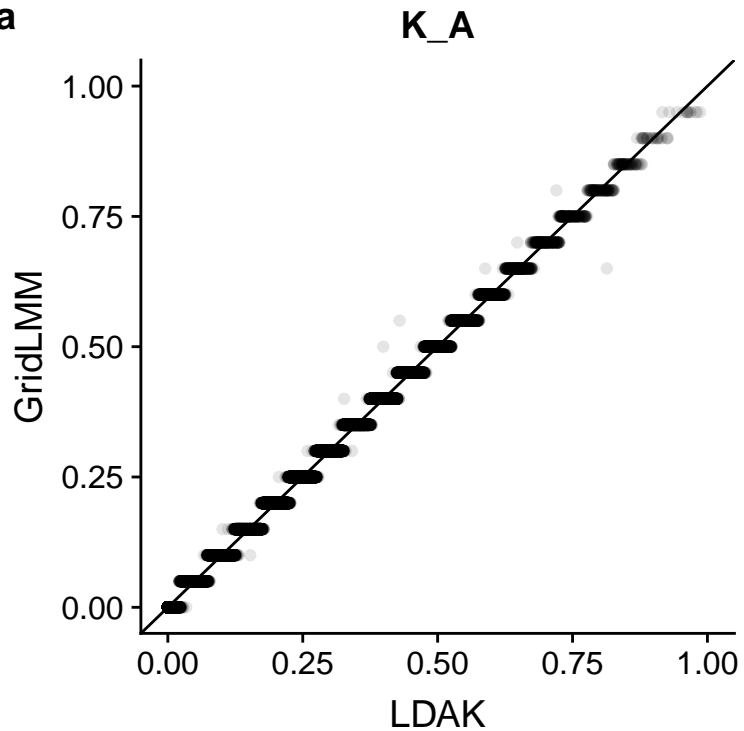**b**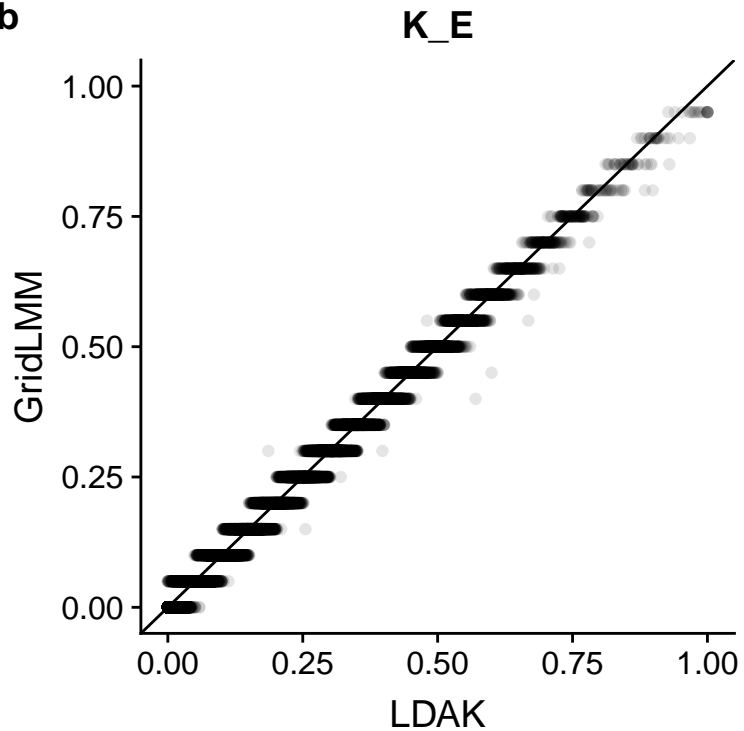

Supplement: S5 Fig — (a) REML estimates for the additive genetic variance (variance component for KA). (b) REML estimates for the epistatic genetic variance (variance component for KE). (PDF) [file pgen.1007978.s005.pdf]

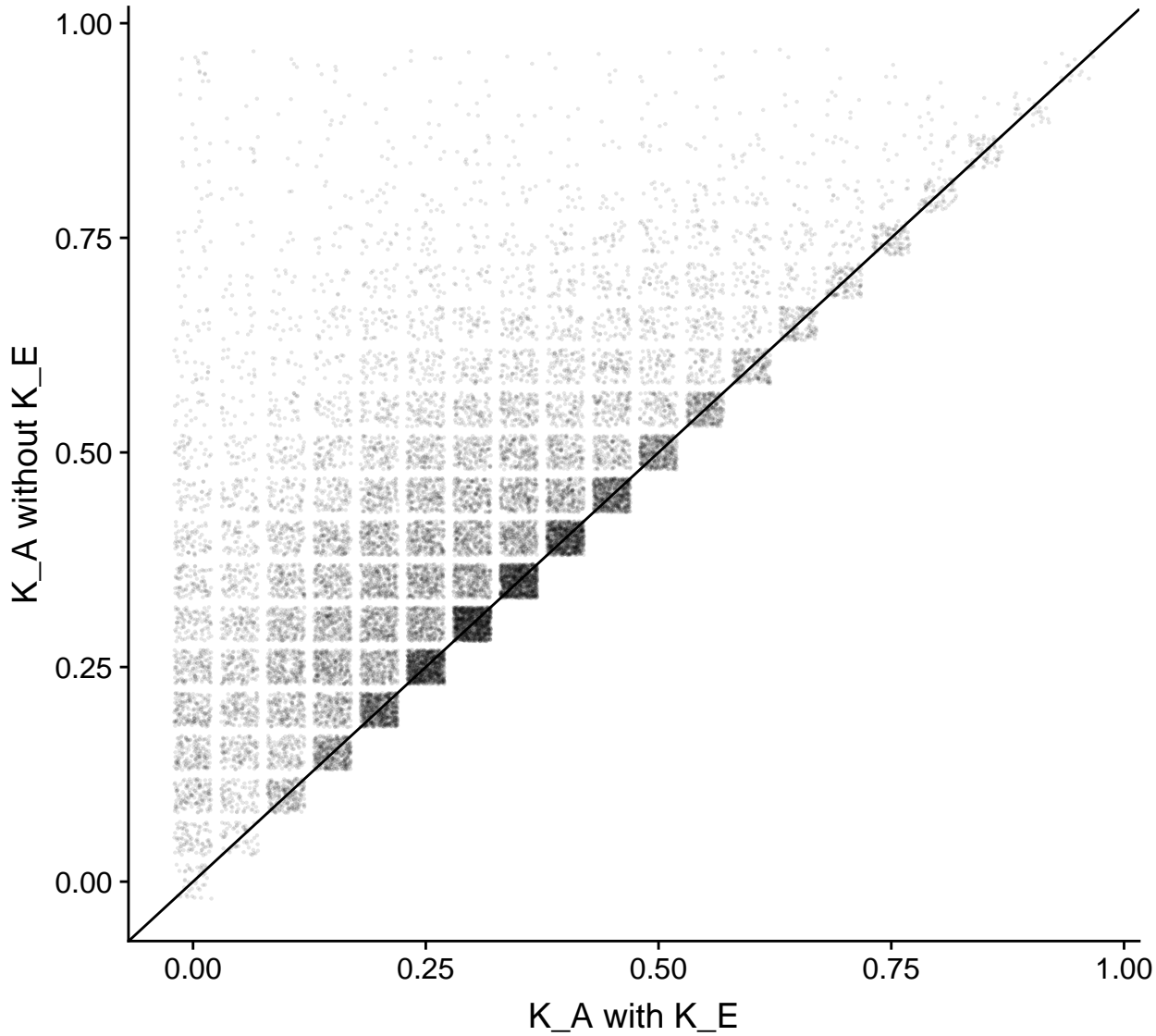

Supplement: S6 Fig — Both models were fit using Grid-LMM with a grid size of 0.05 h2 units. Point positions are jittered for clarity. (PDF) [file pgen.1007978.s006.pdf]

**a****AT1G01820**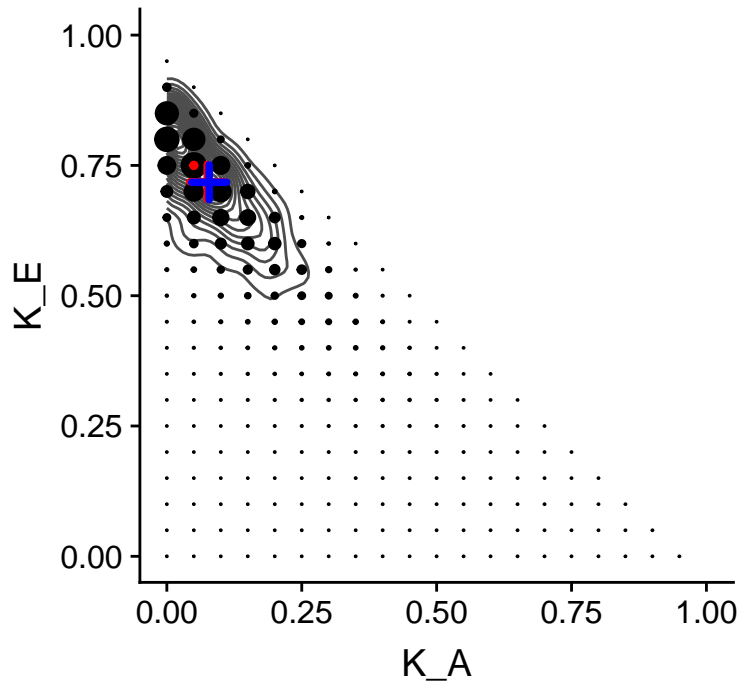**b****AT1G01355**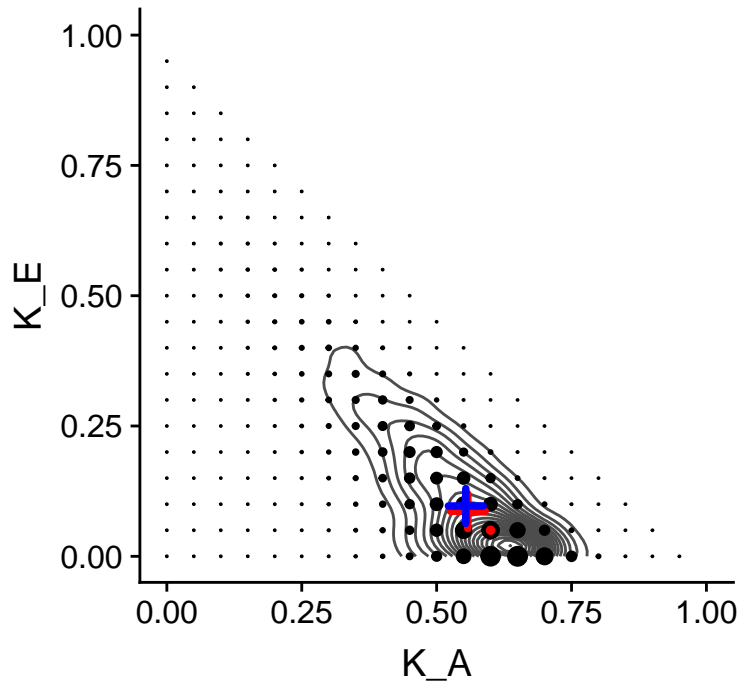

Supplement: S7 Fig — Panels (b) and (c) of Fig 3 in the main text are repeated, except the half-Student-t(3,0,10) prior on the standard deviation of the variance components of KA and KE for the random effects was applied to each grid vertex. The prior was approximated by simulating 1 × 10^4^ independent draws for σA, σE and σe, converting these to prior draws for hA2 and hE2, and then measuring the proportion of draws closest to each grid vertex. (PDF) [file pgen.1007978.s007.pdf]

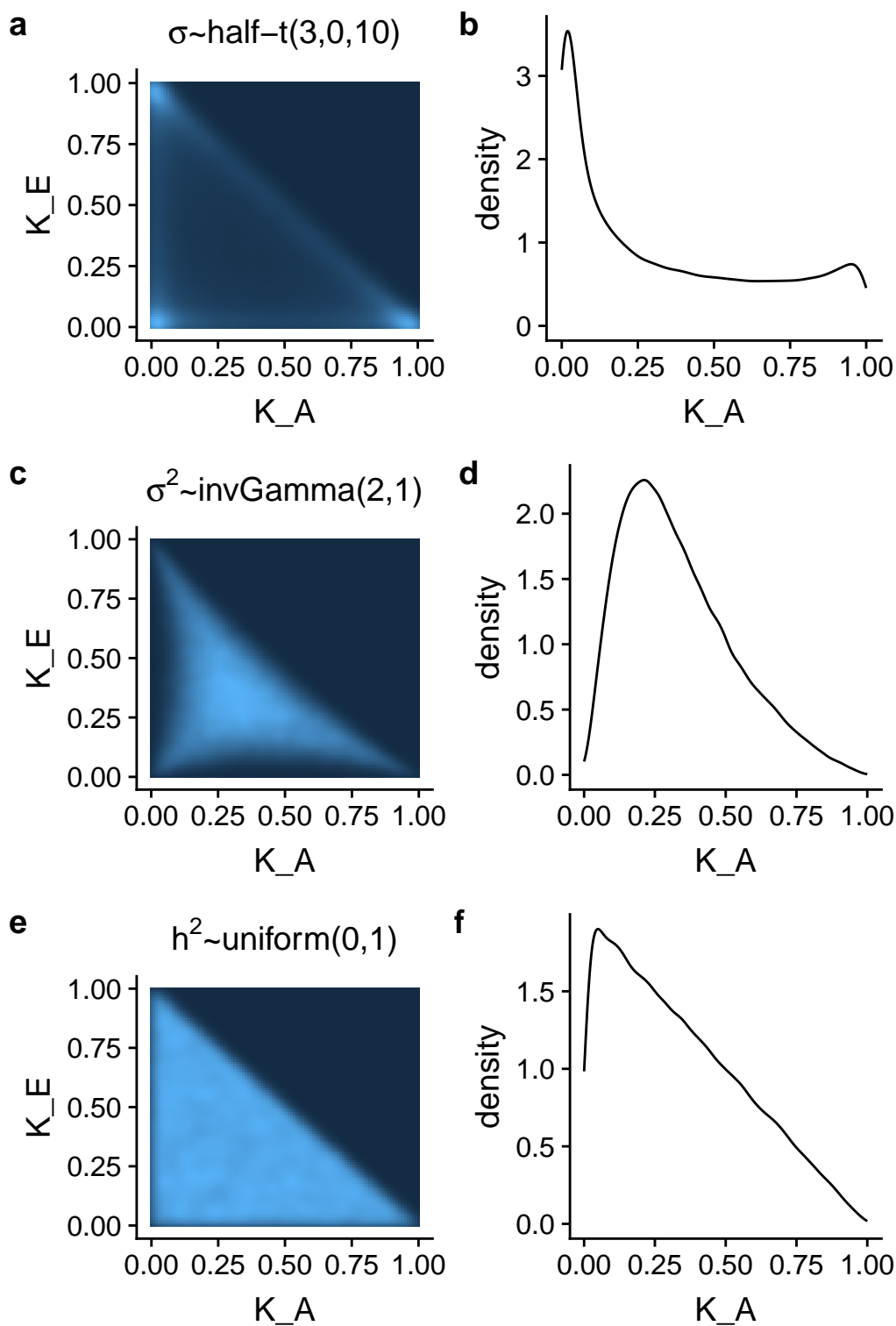

Supplement: S8 Fig — We compare the implied prior distributions on variance component proportions for three classes of priors in a two-random effect model (e.g. KA and KE as random effects plus uncorrelated random error). (a)-(b) Each standard deviation parameter was assigned a half-Student-t prior with 3 degrees of freedom and scale parameter of 10. (c)-(d) Each variance parameter was assigned an inverse-Gamma prior with shape parameter 2 and scale parameter 1. (e)-(g) A uniform prior was applied to the 2-dimensional simplex of [hA2,hE2,he2]. This is the default prior in GridLMM and equivalent to all analyses reported in the main text. (a)-(c)-(e) 2D-density plots for the two variance component proportions. Lighter blue denotes higher prior density. (b)-(d)-(f) Marginal densities for the KA variance component proportion under each prior. The half-Student-t prior implies high probability that only one variance component is important. The inverse-Gamma prior implies high probability that all variance component proportions are non-zero. (PDF) [file pgen.1007978.s008.pdf]
